# Case Report: Fluoroless implantation of left branch bundle pacing in a pregnant patient

**DOI:** 10.3389/fcvm.2023.1325442

**Published:** 2023-12-07

**Authors:** Jun Li, Xiaofang Li, Anxin Zhang, Fanqi Li, Haixiong Wang

**Affiliations:** ^1^ Department of Cardiology, Shanxi Cardiovascular Hospital, Taiyuan, China; ^2^ Department of Digestive Oncology, Shanxi Bethune Hospital, Tongji Shanxi Hospital, Third Hospital of Shanxi Medical University, Shanxi Academy of Medical Sciences, Taiyuan, China; ^3^ Department of Cardiology, The Second Xiangya Hospital, Central South University, Changsha, Hunan, China

**Keywords:** left branch bundle pacing, fluoroless implantation, 3D cardiac model, pregnant patient, pacemaker

## Abstract

A pregnant patient had symptomatic atrial standstill and indications for pacing therapy with an expected high ventricular pacing ratio. With the consideration of potential pacing-induced cardiomyopathy in the future we conducted zero-fluoro left bundle branch pacing (zLBBP) implantation for heart failure prevention. An ex vivo 3D cardiac model (Medtronic, USA) was used preoperatively to simulate the zLBBP implantation to improve procedure safety and efficiency. Intraoperatively, the simulation steps were followed, and a combination of electroanatomic navigation systems (EANS) and intracardiac echocariography (ICE) were used to ensure that the procedure was performed efficiently and safely.

## Brief summary

A pregnant patient showed indications for pacing therapy, with an expected high ventricular pacing ratio. Considering potential pacing-induced cardiomyopathy in the future, we conducted zero-fluoro left bundle branch pacing (zLBBP) implantation for heart failure prevention. An ex vivo 3D cardiac model (Medtronic, USA) was used preoperatively to simulate the zLBBP implantation in order to improve the safety and efficiency of the procedure. Intraoperatively, the simulation steps were followed.

A pregnant patient had symptomatic atrial standstill and indications for pacing therapy, with an expected high ventricular pacing ratio. Considering the potential for pacing-induced cardiomyopathy in the future, we conducted zero-fluoro left bundle branch pacing (zLBBP) implantation for heart failure prevention. An ex vivo 3D cardiac model (Medtronic, USA) was used preoperatively to simulate the zLBBP implantation in order to improve the safety and efficiency of the procedure. Intraoperatively, the simulation steps were followed, and a combination of electroanatomic navigation systems (EANS) and intracardiac echocariography (ICE) were used to ensure that the procedure was performed efficiently and safely.

## Case description

**Figure 1:**
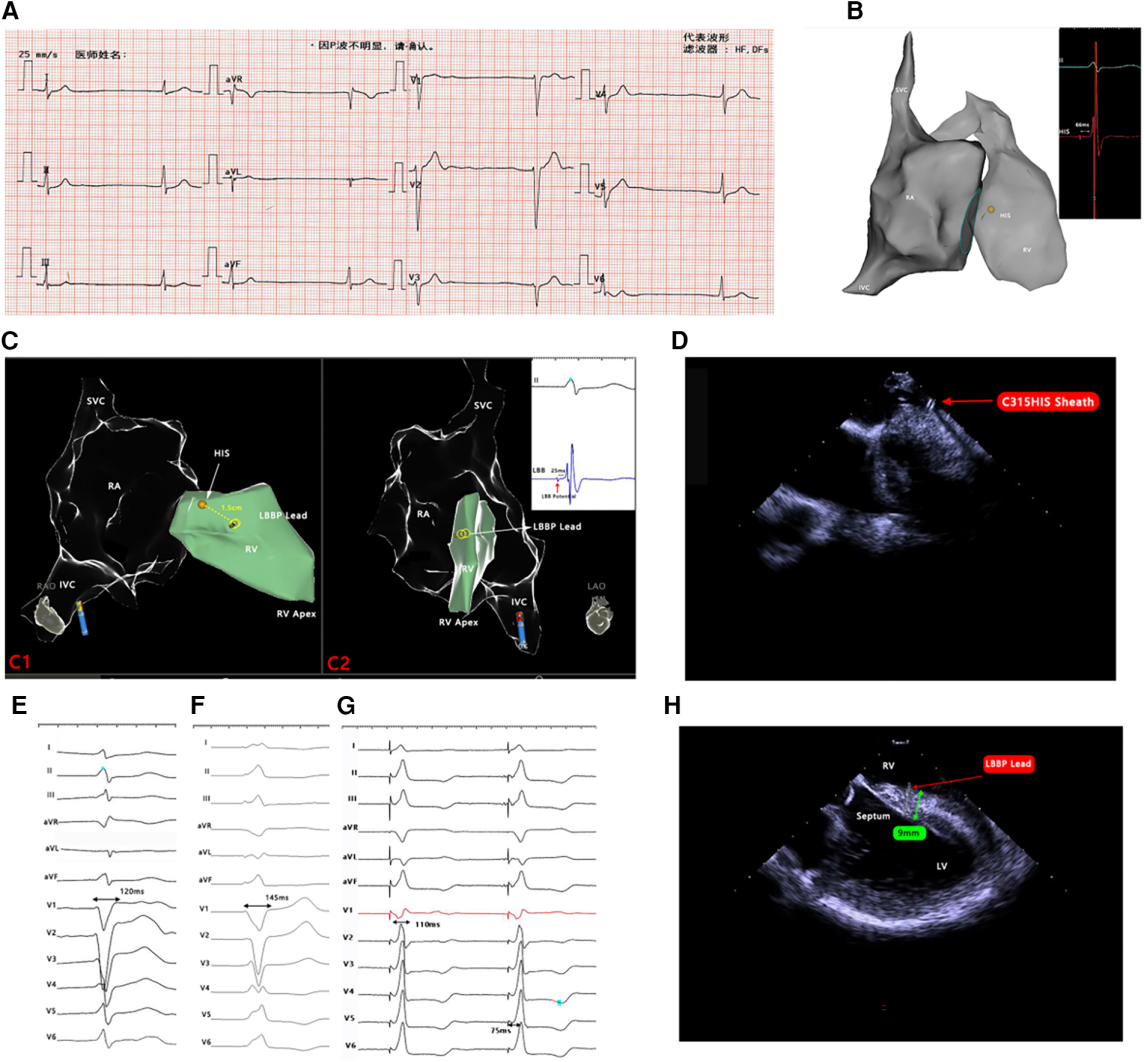
(**A**) Twelve-lead ECG. (**B**) RA and RV models and labeled His potential. (**C**) Models of RA and RV in RAO and LAO positions, the position of the lead tip in relation to the labeled HIS, and the recorded left bundle branch potential. (**D**) The tip of the C315HIS sheath was perpendicular to the septum under ICE. (**E**) Preoperative ECG with a QRS duration of 120 ms. (**F**) RV septal pacing ECG with a QRS duration of 145 ms. (**G**) LBBP ECG with a QRS duration of 110 ms. (**H**) Lead inserted into the septum at a depth of 9 mm under the ICE. ECG, electrocardiogram; RA, right atrium; RV, right ventricle; LBBP, left bundle branch pacing; SVC, superior vena cava; IVC, inferior vena cava; RAO, right anterior oblique; LAO, left anterior oblique; LV, left ventricle, ICE, intracardiac echocardiography.

A 28-year-old female patient experienced intermittent dizziness at 18 weeks of gestation, and an electrocardiogram revealed atrial standstill and junctional escape rhythm (Figure 1A). Cardiac magnetic resonance imaging (CMRI) could not be performed owing to the patient's claustrophobia. The patient showed an indication for pacemaker implantation, with an expected high ventricular pacing ratio.

The final decision was to perform zLBBP. An ex vivo 3D cardiac model was used preoperatively to simulate the procedure. First, we introduced a navigation catheter (Biosense-Webster) in the model to create a 3D electroanatomical map of the superior vena cava (SVC), right atrium (RA), and right ventricle (RV). Then, we simulated the process of zLBBP implantation and sheath removal in the model. The intraoperative procedure, combined with the simulation, is described in the following. The SVC, RA, and RV maps were created in the CARTO3 system using a PentaRay catheter (Biosense-Webster, USA), and His potential was labeled (Figure 1B). Voltage mapping revealed the RA and coronary sinus orifice to be low-voltage zones; furthermore, all RA regions could not be recaptured at 5.0 V/0.5 ms output, which confirmed atrial standstill.

We punctured the subclavian vein and inserted a guidewire into the vein, then connected alligator clips to the CARTO3 system to verify that the guidewire was inserted into the vein. Then, a C315HIS sheath (Medtronic, USA), the most commonly used sheath for left bundle branch pacing (LBBP) implantation, was chosen as the supporting sheath, as the three-dimensional configuration of its tip was very favorable for vertical affixation to the ventricular septum, thus facilitating the screwing of the leads into the myocardium. With the support of the sheath, a 3,830 lead (Medtronic, USA) was delivered into the RV and the lead trajectory was continuously tracked in the CARTO3 system. The lead helix was advanced just outside the sheath and highlighted by connecting alligator clips for visualization in the system by configuring a biopolar catheter in the “catheter setup” menu and placing the pinned ends of two alligator-clip cables connected to the lead in the corresponding pin box locations. Then, the lead tip was positioned 1.5 cm anterior to the labeled His at the line between the HIS and the RV apex in the system (Figure 1C). On ICE visualization, the C315HIS sheath was oriented so that its tip was perpendicular to the septum (Figure 1D), and the lead tip was gradually screwed into the septum. During gradual screwing, the QRS waveform of the lead tip pacing changed gradually from a “W-shape” to an “M-shape” in V1 leads, and the QRS duration changed from 145 ms in RV septal pacing to 110 ms in LBBP (Figure 1E–G). The left bundle branch (LBB) potential was also recorded (Figure 1C), and the pacing stimulus to LV activation time was 75 ms (Figure 1G). The depth of insertion of the lead tip in the septum was 9 mm, as measured by ICE (Figure 1H). The lead pacing thresholds, sensing, and impedance were all normal.

**Figure 2:**
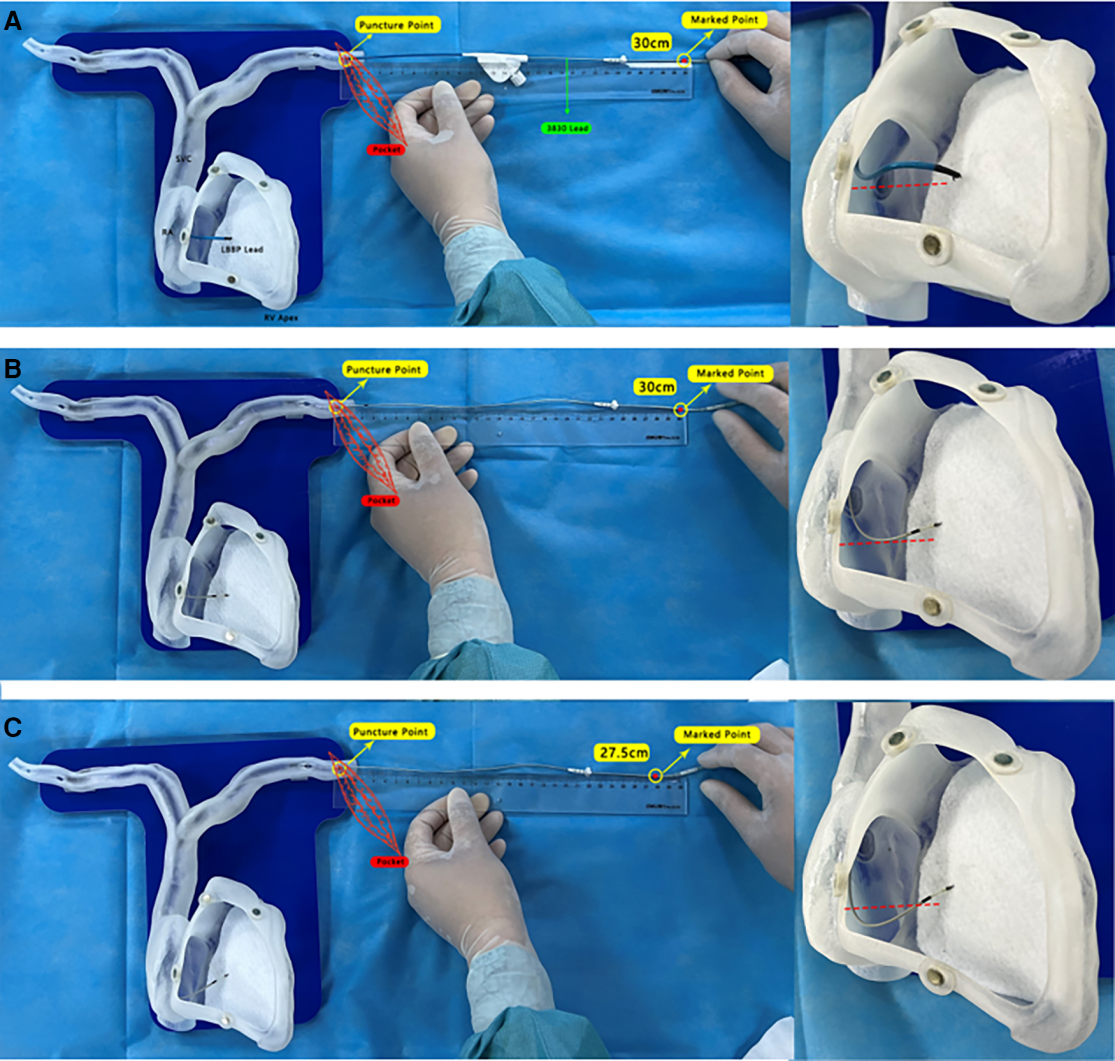
(**A**) The length from the puncture point to the beginning of the thickened 3,830 lead connector was gauged and marked. (**B**) After removing the C315HIS sheath, the length from the puncture point to the lead marked point was measured again, and remained at 30 cm. (**C**) The 3,830 lead was advanced by an additional 2.5 cm (27.5 cm from the puncture point to the marked point), keeping the lead slacked to prevent dislocation.

The length from the puncture point to the beginning of the 3,830 lead connector was assessed and marked (Figure 2A). Then, the C315 sheath was removed and the length from the puncture point to the lead marked point was measured again; it remained unchanged at 30 cm (Figure 2B), ensuring no withdrawal of the deployed lead tip at the LBB. The 3,830 lead was advanced an additional 2.5 cm (27.5 cm from the puncture point to the marked point), with the lead slacked to prevent dislocation (Figure 2C). On ICE visualization, it was further confirmed that the lead position was acceptable, with an appropriate length in the RA without falling into the inferior vena cava. The lead was connected to the IS-1 port of a generator (X3SR01; Medtronic, USA). The procedure took 1 h and 50 min.

## Discussion

LBBP has been shown to be an effective and safe physiological pacing method (1). Usually, pacing leads are implanted using x-ray guidance; however, x-ray exposure may increase the risk of cancer or genetic mutations in exposed individuals. Our pregnant patient had atrial standstill, with an expected high ventricular pacing ratio, which could lead to heart failure. Thus, LBBP was considered be an optimal option for heart failure prevention in this patient. Zero-fluoro procedures should be preferred for special patients such as pregnant women (2). A study of fluoroscopy-free LBBP showed that all device parameters remained stable during follow-up; however, lead slack was suboptimal (too tight or too loose) in some cases (3). This could increase the risk of long-term complications such as lead dislodgement, septum perforation, or an increased capture threshold.

In this article, a novel approach (Figure 2A–C) to ensure optimal lead slack during the zLBBP implantation procedure is presented and validated by *in vitro* model demonstration and intraoperative ICE monitoring. To the best of our knowledge, reports on procedures using the zLBBP implantation approach in pregnant women are rare.

The following key points should be emphasized during the procedure: (1) preoperative simulation of the zLBBP implantation procedure using an ex vivo 3D cardiac model is important in order to shorten the procedure time and to increase the safety of the procedure; (2) it is important to map the His region using the navigation catheter in order to localize the LBB region; (3) on ICE visualization, the C315HIS sheath should be oriented so that its tip is perpendicular to the septum; (4) in zLBBP, it is important to retain the appropriate lead length in the RA to avoid lead dislocation. In this article, a novel approach is presented (Figure 2A–C), which was validated by *in vitro* model demonstration and further confirmed intraoperatively with ICE; and (5) ICE application provides timely monitoring of the depth of lead screwing in order to avoid complications such as perforation.

The patient had no complications such as lead dislodgement, septum perforation, or heart failure during implantation or for 6 months thereafter, and all device parameters remained stable during follow-up.

This case demonstrates that it is safe and feasible to perform zLBBP implantation using EANS for the treatment of special patients such as pregnant women. However, the main limitation of our study is the unavailability of more cases. We look forward to more clinical investigations to further evaluate the effectiveness and long-term effects of this new technology in more patients.

## Data Availability

The raw data supporting the conclusions of this article will be made available by the authors, without undue reservation.
